# Longitudinal survey of knowledge, attitude, and practice of breastfeeding during the COVID-19 pandemic

**DOI:** 10.18332/ejm/203448

**Published:** 2025-04-28

**Authors:** Pattaraporn Ananta, Sirinuch Chomtho, Siriporn Khabuan, Eakkarin Mekangkul, Kamolmart Wannaphahoon, Duangporn Maitreechit, Sophie Gallier, Orapa Suteerojtrakool

**Affiliations:** 1 Division of Nutrition, Department of Pediatrics, King Chulalongkorn Memorial Hospital, Bangkok, Thailand; 2 Center of Excellence in Pediatric Nutrition, Division of Nutrition, Department of Pediatrics, Faculty of Medicine, Chulalongkorn University, Bangkok, Thailand; 3 Lactation Clinic, King Chulalongkorn Memorial Hospital, Bangkok, Thailand; 4 Science Department, Dairy Goat Co-operative (N.Z.) Ltd, Hamilton, New Zealand; 5 Ambulatory Division, Department of Pediatrics, King Chulalongkorn Memorial Hospital, Bangkok, Thailand

**Keywords:** knowledge, attitude, practice, COVID-19 pandemic, predominant breastfeeding

## Abstract

**INTRODUCTION:**

Concerns about viral transmission through breast milk may impact breastfeeding recommendations and success. This study examined the changes in maternal knowledge, attitudes, and practices (KAP) related to breastfeeding and COVID-19 transmission during the pandemic.

**METHODS:**

A longitudinal survey of Thai mothers was conducted when infants were 2, 12, and aged 24 weeks (March 2022–April 2023). The questionnaire, assessing knowledge of SARS-CoV-2 transmission, attitudes toward breastfeeding, and hygiene practices, was validated using the Item-Objective Congruence index. Good KAP was defined as scoring >60% in each section. Associated factors, including maternal age, education level, occupation, breastfeeding experience, and history of recent COVID-19 illness, were analyzed using multivariable linear regression.

**RESULTS:**

A total of 195 mothers (mean age: 30.6 ± 6 years) completed the survey. One-third had good KAP, with an increasing trend over 24 weeks. Knowledge and practice scores among mothers without a recent history of COVID-19 illness demonstrated a positive change at an infant age of 24 weeks (95% CI: 0.08–0.96 and 0.02–0.76, respectively), while attitude scores remained unchanged. Maternal age at delivery was positively associated with the attitude changes (β=0.19; 95% CI: 0.02–0.20), whereas maternal education level and monthly family income were negatively associated. Better knowledge and attitudes about COVID-19 transmission during breastfeeding at 2 and 12 weeks were linked to a higher likelihood of predominant breastfeeding at 24 weeks postpartum.

**CONCLUSIONS:**

Maternal knowledge, attitudes, and practices (KAP) improved over 24 weeks, with significant gains in knowledge and practice among mothers who had not recently contracted COVID-19. Early knowledge and attitude were linked to sustained predominant breastfeeding, highlighting the importance of targeted education.

**CLINICAL TRIAL REGISTRATION:**

The study was part of the study entitled ‘Infant feeding survey during COVID-19 pandemic’ registered on the official website of Thaiclinicaltrials.org

**IDENTIFIER:**

ID TCTR20220215012

## INTRODUCTION

Breastfeeding is a crucial strategy to promote infant health. Breast milk contains optimal nutrients, hormones, and bioactive agents that support infant growth and development. Extensive studies have shown that exclusively breastfed infants have a lower risk of infectious diseases, obesity, and type I diabetes mellitus than infants receiving partial breastfeeding or infant formula^1^. Previous studies have also found that breastfed infants have a higher IQ score than those who were not breastfed^2^.

Since the benefit of breastfeeding is well established, the World Health Organization (WHO) recommends that infants be exclusively breastfed for the first six months and continue with appropriate complementary food for up to two years of age^3^. Although the data from previous studies show the advantages of breastfeeding, the rate of exclusive breastfeeding in Thailand is still low. The Multiple Indicator Cluster Survey (MICS) reports of 2019 in Thailand showed that only 14% of infants aged 0–5 months were exclusively breastfed^4^, and increased to 28.6% in 2022^5^. These numbers are still far below the global target of 50% exclusive breastfeeding in 2025.

Many studies explore the barriers to exclusive breastfeeding^3,6,7^. Previous evidence shows that maternal knowledge and attitudes toward exclusive breastfeeding are important factors affecting the maternal decision on the duration of breastfeeding and the types of infant feeding^6^. Furthermore, other factors, including breast problems (e.g. mastitis, sore nipples, and breast abscess), maternal education level, socioeconomic status, and influence from family members may affect the maternal decision to practice exclusive breastfeeding^6^. Moreover, the concern of breast milk safety during maternal infection is also a determinant influencing maternal decision-making on infant feeding practice^6,7^.

COVID-19 is a respiratory disease caused by coronavirus, namely SAR-CoV-2^8-10^. The potential impact on breastfeeding practice arose from the concerns regarding viral transmission through breast milk among lactating mothers, especially among mothers who had COVID-19 illness^6^. Previous studies about the impact of the COVID-19 pandemic on breastfeeding practice showed that the COVID-19 pandemic had negatively affected breastfeeding rates. Prior research conducted in Italy revealed that during the COVID-19 pandemic, there was a notable increase in the utilization of infant formula among postpartum women who had delivered either prior to or during the pandemic. Conversely, there was a noteworthy reduction in exclusive breastfeeding rates, from 74.2% to 32.8%^11^. Nevertheless, previous findings indicated that breastfed infants might acquire antibodies against SARS-CoV-2 from their mothers who have received the COVID-19 vaccinations or previously had a COVID-19 infection^12-16^.

Few studies explore the knowledge, attitudes, and practices (KAP) of breastfeeding, especially in regard to preventive measures during the COVID-19 outbreak. Therefore, our study aimed to investigate differences in within-participant changes in breastfeeding KAP concerning COVID-19 over the first 6 months of life and compare parents with and without a recent history of COVID-19 illness among Thai parents and caregivers.

## METHODS

### Study design and study population

A longitudinal survey was conducted from March 2022 to April 2023, when Thailand was still under the COVID-19 pandemic influence with ongoing infections and hygiene measures in place. This sub-study was part of the study entitled ‘Infant feeding survey during COVID-19 pandemic’ (Thai Clinical Trials Registry: TCTR20220215012).

Thai mother–infant dyads, starting from the infant age of 2 weeks until 6 months old, were enrolled from the postpartum ward of the King Chulalongkorn Memorial Hospital, Bangkok, Thailand, and its social networks. The inclusion criteria were mothers of healthy full-term infants with a birth weight of 2.5–4.5 kg, a singleton neonate, and an age of less than 2 weeks at enrollment. We excluded mothers of infants with any disorders that may interfere with nutrition, growth, or development of the immune system.

The sample size was calculated using the Taro-Yamane method. According to data from the Ministry of Public Health of Thailand, there were approximately 500000 pregnant women in 2022. Hence, to adequately represent this target population with a precision level of ±10% and a confidence level of 95%, a minimum of 100 participants was required.

This study was approved by the Institutional Review Board, Faculty of Medicine, Chulalongkorn University (IRB No. 956/64). Parents/guardians of the infants participating in this study provided informed consent following a detailed explanation of all study procedures by the researchers.

### Data collection

Demographic data, including infant details (age, sex, birth weight, mode of delivery, postnatal complications), maternal history (age at delivery, pregnancy complications, occupation, maternal education level, length of maternity leave, breastfeeding intention, and breastfeeding experience), infant feeding, barriers to breastfeeding, and family socioeconomic status were collected using a self-administered questionnaire. Subsequently, interviews were conducted by the research assistants to verify all the answers to the questionnaires.

### Definitions

*Predominant breastfeeding infant*

Defined as an infant whose main source of nutrition was breastmilk and who received infant formula less than 12 days between birth and six months of age. No liquid or solids are given except for water, oral hydration solution, or drops/syrups of vitamins, minerals, or medicines before 4 months of age.

*Mother with a recent history of COVID-19 infection*

Defined as a mother who was infected with SARS-CoV-2 virus (confirmed by antigen test kit or polymerase chain reaction) in the past 6 months.

### Knowledge, attitude, and practice of breastfeeding

The evaluation of knowledge, attitude, and practice of breastfeeding was conducted using a questionnaire that encompassed three distinct sections:

1. The knowledge section included nine questions, allowing participants to select responses from options, such as ‘neutral’, ‘uncertain’, and ‘disagree’. The total possible score for this section was up to 9 points.
2. The attitude section comprised 14 questions, providing participants with response choices of ‘neutral’, ‘uncertain’, and ‘disagree’. The maximum achievable score for this section was 14 points.
3. The practical section consisted of three questions, offering response options of ‘always’, ‘usually’, ‘rarely’, and ‘never’. The highest possible score for this component was 6 points.

The details of the questionnaires and the scoring criteria are given in Supplemental file Table 1.

Before the questionnaire was applied, it was verified using the Item-Objective Congruence (IOC) index by five pediatric nutrition specialists. Any items of the questionnaire that had IOC scores <0.5 were revised accordingly. The criteria for assessing good KAP were determined based on attaining correct answers >60% of the maximum scores.

The survey was prospectively conducted through interviews of caregivers via phone or teleconference platform by the research team at 2, 12, and 24 weeks of age. The tolerance limits for follow-up were ± 3 days for the age of 2 weeks, and ± 5 days for the ages of 12 and 24 weeks. The data collection and management were conducted using REDCap electronic data capture tools hosted at Chula Data Management Centre, Faculty of Medicine, Chulalongkorn University.

The success of predominant breastfeeding at 24 weeks was analyzed in relation to maternal knowledge and attitude scores on breastfeeding during the COVID-19 pandemic, as assessed through the questionnaires, providing insight into the pandemic’s potential influence on breastfeeding practices.

### Statistical analysis

The statistical analyses were performed using SPSS version 26 (SPSS Inc., Chicago, IL, USA). The data were checked for their normality by using a histogram and Kolmogorov-Smirnov test before analysis. The continuous data, including demographic data and KAP scores, are presented as mean ± standard deviation (SD) or median and interquartile range (IQR), as appropriate. Independent samples t-test, Mann-Whitney U test, and chi-squared test or Fisher’s exact test were used to compare and analyze the demographic data between two groups (mothers with and without a history of COVID-19 infection). Repeated measures ANOVA was used to explore the changes in KAP scores in all lactating mothers at different time points. Bonferroni test was performed to compare the changes in KAP scores between mothers with a recent history of COVID-19 illness and mothers with no history of COVID-19 illness.

Multivariable linear regression analysis was performed to explore the factors associated with the changes in KAP scores. The variable factors derived from previous literature reviews (maternal age, education level, occupation, breastfeeding experience, and maternal history with COVID-19 infection) were applied to adjust the model. In addition, univariate logistic regression was performed before binary logistic regression to explore the association between KAP scores and the success of predominant breastfeeding until 6 months of the infant’s age. The adjusted factors for binary logistic regression were maternal knowledge scores at 2 and 12 weeks, positive attitude scores at 12 weeks, maternal education level, breastfeeding experience, and maternal age at delivery. All statistical tests were 2-sided, and p<0.05 was considered statistically significant. Moreover, survival analysis was performed to explore the probability of successful predominant breastfeeding until 24 weeks of infant age.

## RESULTS

**Figure 1:**
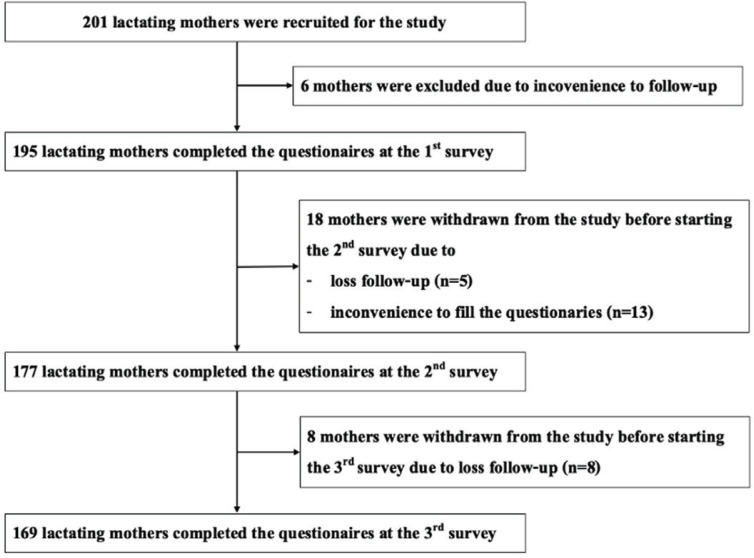
Recruitment flow diagram, a longitudinal survey during COVID-19 pandemic of Thai mothers, conducted at infant age of 2,12, and 24 weeks, Bangkok, March 2022–April 2023

A total of 195 participants completed the initial survey, while 169 remained for the final survey conducted at 24 weeks of infant age. Most of the drop-out participants cited inconvenience in filling in or responding to the questionnaires (Figure 1).

The average age of the lactating mothers was 30.6 ± 6 years. Around 42% of the mothers were first parity, nearly half of the mothers had obtained a Bachelor’s degree or higher, and half were employed in an office or had a similar occupation. Approximately 34% of the mothers reported that they belonged to the low- to middle-income family, with monthly income of around 440–870 US$ (Table 1).

**Table 1:** Baseline characteristics at the first survey of the study (N=195)

*Characteristics*	*n (%)*
**Maternal age at delivery** (years), mean ± SD	30.6 ± 6.0
**Maternal education level**	
Primary school or lower	11 (5.7)
Secondary school	56 (28.7)
Commercial/vocational school	36 (18.5)
Bachelor’s degree	73 (37.4)
Master’s degree	16 (8.2)
Professional or doctorate	3 (1.5)
**Paternal education level**	
Primary school or lower	15 (7.7)
Secondary school	69 (35.4)
Commercial/vocational school	41 (21.0)
Bachelor’s degree	54 (27.7)
Master’s degree	14 (7.2)
Professional or doctorate	2 (1.0)
**Maternal occupation**	
Business owner/self-employed	27 (13.8)
Healthcare professional	13 (6.7)
Government/civil services	4 (2.1)
Manager/supervisor	1 (0.5)
Office/employee	98 (50.3)
Not working	44 (22.6)
Others	8 (4.0)
**Paternal occupation**	
Business owner/self-employed	53 (27.2)
Healthcare professional	7 (3.6)
Government/civil services	10 (5.1)
Office/employee	113 (58.0)
Educator	1 (0.5)
Not working	4 (2.0)
Others	7 (3.6)
**Monthly family income (US$)**	
<440	32 (16.4)
440–880	67 (34.4)
>880–1470	46 (23.6)
>1470–2930	32 (16.4)
>2930	18 (9.2)

### Overall knowledge, attitude, and practice concerning breastfeeding during the COVID pandemic

The participants’ knowledge, attitude, and practice scores exhibited a tendency to increase over time, from 2 to 24 weeks of infant age, as demonstrated in Table 2. The analysis using repeated measures ANOVA demonstrated significant changes in KAP scores. The knowledge and practice scores significantly increased from 2 to 24 weeks of infants’ age (4.82 ± 1.88 vs 5.05 ± 1.86, p=0.005; 2.55 ± 1.29 vs 2.94 ± 1.37, p=0.001; respectively). However, the positive attitude showed an increasing trend, although it was not statistically significant.

**Table 2:** Knowledge, attitude, and practice (KAP) scores of breastfeeding

*Scores^a^*	*Age 2 weeks* *(N=195)* *Mean ± SD*	*Age 12 weeks* *(N=177)* *Mean ± SD*	*Age 24 weeks* *(N=169)* *Mean ± SD*	*p* *within* *group^b^*	*Change from 2 to* *12 weeks* *(95% CI)*	*Change from 2* *to 24 weeks* *(95% CI)*
**Knowledge**	4.82 ± 1.88	5.05 ± 1.86	5.29 ± 2.17	0.005	0.27 (-0.03–0.56)	0.45 (0.09–0.82)
**Attitude**	7.89 ± 2.92	8.12 ± 3.27	8.29 ± 3.38	0.55	0.27 (-0.39–0.93)	0.18 (-0.47–0.84)
**Practice**	2.55 ± 1.29	2.94 ± 1.37	2.97 ± 1.31	0.001	0.30 (-0.01–0.61)	0.43 (0.14–0.73)

a Breastfeeding KAP was assessed using a structured questionnaire consisting of three sections. The knowledge section included nine questions with response options such as ‘neutral’, ‘uncertain’, and ‘disagree’, with a maximum score of 9 points. The attitude section comprised 14 questions with the same response options, allowing a maximum score of 14 points. The practice section contained three questions with response choices of ‘always’, ‘usually’, ‘rarely’, and ‘never’, with a maximum score of 6 points.

b Within-group differences in mean scores were analyzed using repeated measures ANOVA, while changes over time were assessed using the Bonferroni test.

### Comparison of the knowledge, attitude, and practice scores between mothers with and without a recent history of SARS-CoV-2 infection

The demographic data of mothers with and without a recent history of COVID-19 infection were analyzed to determine the differences between the two groups; the results showed that there were no significant variations in the demographic data (data not shown).

**Figure 2:**
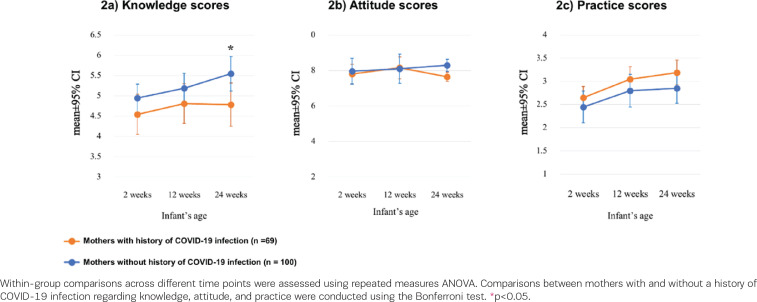
Comparison of the changes in knowledge, attitude, and practice scores

Figure 2 shows that the knowledge scores of mothers without a recent history of SARS-CoV-2 infection were higher than those with a recent history of SARS-CoV-2 infection at 24 weeks (5.59 ± 2.18 vs 4.83 ± 2.11, p=0.03). However, no significant differences regarding attitude and practice scores were found between the two groups at 2, 12, and 24 weeks of infant age.

### Factors associated with change in KAP scores from 2 to 24 weeks of infant age

A multivariate analysis was employed to investigate the factors related to the changes in knowledge, attitude, and practice (KAP) scores (Supplement file Tables 2–4). The findings revealed that older maternal age at delivery was linked to a smaller increase in knowledge scores between 2 and 12 weeks postpartum, with a β coefficient of -0.18 (95% CI: -0.10 – -0.01). Conversely, mothers with a professional or doctorate degree showed a greater increase in knowledge scores over the same period (β=0.17; 95% CI: 0.19–8.53).

Maternal age at delivery displayed a positive association with attitude scores from 12 to 24 weeks (β=0.19; 95% CI: 0.02–0.20). However, compared to mothers who completed only primary school, those with a higher education level showed smaller increases in attitude scores from 12 to 24 weeks postpartum. This included mothers with a commercial/vocational school background (β= -0.24, 95% CI: -4.45–0.11), a Bachelor’s degree (β= -0.41, 95% CI: -5.34 – -0.78), and a Master’s degree (β= -0.36; 95% CI: -8.11 – -1.72). Higher family income was associated with lower attitude scores. Moreover, the results showed that the change in practice scores from 2 to 12 weeks was negatively associated with middle-level monthly family income (>880–1470 US$) compared with low monthly income (β= -0.23, 95% CI: -1.97 – -0.10).

### Association between KAP scores and the success of predominant breastfeeding until 6 months

**Figure 3:**
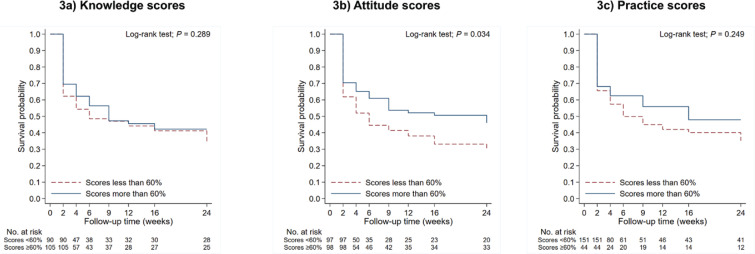
Survival probability of the predominant breastfeeding between mothers with good knowledge, attitude, and practice scores (>60%) and not good scores (<60%) (N=169)

At the last follow-up conducted when the infants reached 24 weeks of age, a total of 169 participants remained actively engaged in the survey. The result shows that only one-third of the lactating mothers were able to practice predominant breastfeeding until 24 weeks of infant age during the COVID-19 pandemic. Among 69 lactating mothers with recent COVID-19 infection, 44% reported stopping breastfeeding due to their concerns about viral transmission through breast milk. Figure 3 shows a survival analysis exploring the probability of success in predominant breastfeeding. The results revealed that mothers with good attitude scores (>60%) had a higher probability of success in predominant breastfeeding until 6 months, compared to the group of mothers with attitude scores <60%, out of the maximum scores (p=0.034). Although there were no significant differences in successful breastfeeding rate between the mothers who had good practice scores and those with lower scores, it was observed that the probability of successful breastfeeding for mothers with good practice scores tended to result in longer breastfeeding durations.

The relationship between KAP scores and the successful practice of predominant breastfeeding until the infant reached 6 months is presented in Table 3. The results indicated that a better knowledge of COVID-19 transmission at 2 weeks (OR=1.22; 95% CI: 1.02–1.46) and at 24 weeks of the infant’s age (OR=1.24; 95% CI: 1.07–1.45) were positively associated with the likelihood of achieving predominant breastfeeding at 6 months. Additionally, mothers who exhibited positive attitude scores at infant age of 12 weeks were also more likely to succeed in practicing predominant breastfeeding at 6 months (OR=1.19; 95% CI: 1.07–1.32).

**Table 3:** Factors associated with the success of predominant breastfeeding at 6 months (N=169)

*Factors*	*OR^a^*	*95% CI*	*p*
**Maternal age at delivery**	1.04	0.99–1.09	0.16
**Knowledge score**			
At 2 weeks	1.22	1.02–1.46	0.03*
At 12 weeks	1.17	0.98–1.39	0.09
At 24 weeks	1.24	1.07–1.45	0.01*
**Positive attitude score**			
At 2 weeks	1.09	0.98–1.22	0.11
At 12 weeks	1.19	1.07–1.32	0.01*
At 24 weeks	0.96	0.76–1.21	0.74
**Practice score**			
At 2 weeks	0.95	0.74–1.22	0.68
At 24 weeks	1.06	0.84–1.34	0.64
**Mother’s education level**			0.19
Primary school or lower ®	1		
Secondary school	1.97	0.36–10.78	0.44
Trade/vocational school	0.81	0.13–4.91	0.82
Bachelor’s degree	2.38	0.45–12.76	0.31
Master’s degree	3.86	0.59–25.29	0.16
Professional or doctorate	1.50	0.08–26.86	0.78
**Monthly family income** (US$)			0.86
<440 ®	1		
440–880	0.89	0.35–2.23	0.80
>880–1470	0.96	0.35–2.59	0.93
>1470–2930	1.16	0.40–3.31	0.79
>2930	1.64	0.48–5.62	0.43
**Maternal occupation**			
Not working ®	1		
Working	0.79	0.37–1.68	0.54
**Breastfeeding experience**			0.15
No ®	1		
Yes	1.58	0.85–2.95	0.15
**Maternal history of COVID-19 infection** ^a^			0.94
No ®	1		
Yes	1.02	0.54–1.93	0.94

a The total number of mothers who had history of COVID-19 infection, n=69.

* p<0.05.

® Reference categories.

## DISCUSSION

This study was the first longitudinal survey of KAP regarding breastfeeding practice in Thailand during the COVID-19 pandemic. The primary objective was to assess within-participant changes in breastfeeding KAP concerning COVID-19 during this pandemic and to explore its association with the success of breastfeeding until 6 months of infant age.

Our study revealed that maternal knowledge and practice scores about breastfeeding during the COVID-19 pandemic gradually increased as their infants grew older. One possible explanation could be that, over time, mothers had more opportunities to access reliable information about breastfeeding and infection control, particularly during their postpartum or maternity leave period. As they adjusted to motherhood, they may have become more engaged in seeking out guidance and health information, leading to improved understanding and confidence in breastfeeding practices. However, our findings were different from previous studies in Nepal^17^ and Pakistan^18^, which showed that postpartum mothers had good knowledge and practice towards preventive measures related to the COVID-19 transmission. This discrepancy could be due to the study design and the difference from country to country, and the researchers did not provide information on breastfeeding. In addition, a study among 389 Syrian refugee mothers showed that despite having good knowledge about COVID-19 transmission through social media, they still lacked knowledge about transmission of COVID-19 between mothers and child, possibly due to limited available information^19^.

Our study observed the differences in knowledge scores between the lactating mothers with a recent history of SARS-CoV-2 infection and those without such history, with higher scores recorded in the latter group during the 12 to 24 weeks of infant age. Furthermore, our findings also demonstrated that the change in knowledge scores were significantly higher in the group of mothers without a recent history of COVID-19 infection compared to those experiencing a recent infection. The plausible explanation could be that the mothers who were unaffected by SARS-CoV-2 may have accessed available information on COVID-19, equipping them with comprehensive knowledge about the virus and effective preventive measures, and getting more knowledgeable, thus preventing them from COVID-19. On the other hand, data collection was done while the lactating mothers may have experienced strict measures towards COVID-19; therefore, the attitude and practice may remain steady over the first six months of lactation.

Intriguingly, the findings of the present study indicated that higher maternal education level and family income were significantly associated with a decrease in attitude scores over time. One possible reason for the observation might be that these mothers might have had great concerns about the COVID-19 situation, including viral transmission, since the beginning of the survey, which amplified their worries regarding the safety of breastfeeding. In addition, due to the variation in social awareness, communication, and distribution of information about the COVID-19 outbreak and the SARS-CoV-2 virus in each time period, the mothers may have a higher fear of contracting the SARS-CoV-2 virus at a time of a more serious situation.

More importantly, our findings indicated that the higher knowledge scores at 2 and 24 weeks and the positive attitude at 12 weeks of infant age resulted in a high likelihood of the success of predominant breastfeeding until the infants reached 24 weeks old. Additionally, the findings from the survival analysis revealed a substantial likelihood of successful predominant breastfeeding among mothers who displayed favorable attitude scores, signifying a coherent alignment in the same direction. Although statistical significance was not achieved, the median survival time among mothers with commendable practice scores indicated a prolonged duration of predominant breastfeeding in comparison to those whose scores were not considered favorable. These findings were consistent with previous studies. For instance, the recent community-based cross-sectional study showed that the mothers who were in the higher knowledge category had a higher likelihood of exclusive breastfeeding practice during the COVID-19 pandemic in Ethiopia^20^. Likewise, mothers with positive or desired attitudes showed more than twice as high odds of EBF compared to those with less positive or undesired attitudes. Moreover, a previous study in Bangkok, Thailand, by Topothai et al.^21^ showed that mothers with the intention to breastfeed for a longer duration (≥6 months), higher education level, higher income level, breastfeeding experience, and exposure to breastfeeding advice during pregnancy were more likely to successfully practice six-month exclusive breastfeeding. Compared to prior the pandemic, the factors associated with success of predominant breastfeeding were similar during the pandemic^22^. However, the COVID-19 pandemic introduced an additional concern – among mothers who were infected, the fear of transmitting the virus to their infants became a significant and novel driver for switching to formula feeding. Therefore, providing accurate information about SARS-CoV-2, along with regular breastfeeding counseling, remains essential for enhancing maternal attitudes and knowledge on breastfeeding, particularly during pandemics.

To the best of our knowledge, this is the first longitudinal survey conducted in Thailand and South-East Asia that explored the KAP of breastfeeding during the global health crisis caused by SARS-CoV-2. The study also investigated the factors associated with the change in KAP scores and the relationship between the KAP of breastfeeding and the success of predominant breastfeeding.

### Limitations

This study has some limitations. The participants were recruited exclusively from Bangkok, Thailand, during the late phase of the COVID-19 pandemic, which may limit the generalizability of the findings to other regions or different stages of the pandemic. Additionally, the reliability of the KAP questionnaire was not assessed, which may impact the robustness of the measurement. Furthermore, the study did not assess the level of fear related to COVID-19, which could have influenced maternal KAP. While key demographic factors such as maternal age, infant age, feeding mode, socioeconomic status, and history of COVID-19 were considered, the potential for residual confounding remains, as unmeasured factors such as health literacy, social support, or cultural influences may have influenced the results.

## CONCLUSIONS

Over the first six months postpartum, maternal KAP concerning COVID-19 transmission during breastfeeding demonstrated a significant improvement, with notable gains in knowledge and practice, particularly among mothers without a recent history of COVID-19. Furthermore, higher levels of knowledge and positive attitudes in the early postpartum period were associated with a greater likelihood of sustaining predominant breastfeeding at 24 weeks. These findings emphasize the importance of targeted educational interventions and ongoing support to enhance breastfeeding practices, particularly in the context of public health crises.

## Supplementary Material



## Data Availability

The data that support the findings of this study are available from the corresponding author upon reasonable request.
