# Supplementary material for: Longitudinal survey of knowledge, attitude, and practice of breastfeeding during the COVID-19 pandemic
Source: Eur J Midwifery. 2025 Apr 28;9:10.18332/ejm/203448. doi: 10.18332/ejm/203448 (PMC12527125; doi:10.18332/ejm/203448)
Supplement: Supplementary file 1 [file EJM-9-21-s1.pdf]

Supplemental Table 1 Scoring criteria for KAP questionnaires[1,2]

Knowledge part (Total scores = 9; Corrected answer = 1 point)	Neutral (point)	Uncertain (point)	Disagree (point)
Can COVID-19 be transmitted from the mother to the baby during pregnancy?	-1	0	1
Can COVID-19 be transmitted from the mother to the baby through breast milk?	-1	0	1
Can COVID-19 spread through respiratory droplets from a COVID-19-positive case?	1	0	-1
To prevent the spread of COVID-19, should people avoid going to a community mall and using public transport?	1	0	-1
Are COVID-19 vaccinations safe for pregnant women and their babies?	1	0	-1
Can COVID-19 vaccinations reduce severe illness from COVID-19 infection?	1	0	-1
Are COVID-19 vaccinations safe for breastfeeding mothers and their babies?	1	0	-1
After the breastfeeding mother receives the COVID-19 vaccine, can the immunity transfer to the baby through breast milk?	1	0	-1
Is formula feeding safer than expressed breast milk if the mother has a COVID-19 infection?	-1	0	1
**Attitude part (Total scores = 14; 1 point = positive attitude)**	**Neutral (point)**	**Uncertain (point)**	**Disagree (point)**
Breastfeeding is the best option for infant feeding during the COVID-19 pandemic.	1	0	0
Is it safer to switch to formula milk during the COVID-19 pandemic?	0	0	1
I am afraid of transferring COVID-19 to my baby through breastfeeding.	0	0	1
If I had a COVID-19 infection, I would stop breastfeeding.	0	0	1
Wearing a surgical mask ± face shield during breastfeeding can reduce the risk of COVID-19 transmission from the mother to the baby.	1	0	0
Strict hand washing before breastfeeding can reduce the risk of COVID-19 transmission from the mother to the baby.	1	0	0
I would continue breastfeeding despite having a COVID-19 positive case in the household.	1	0	0
I would continue breastfeeding after receiving COVID-19 vaccinations.	1	0	0
Side effects of COVID-19 vaccinations may prevent me from continuing breastfeeding.	0	0	1
It is safer to switch to formula feeding after I have COVID-19 vaccinations.	0	0	1
I want to be vaccinated and breastfeed to provide immunity to my baby.	1	0	0

I do not want visitors because they might have COVID-19 and can infect me and/or my baby.	1	0	0
Breastfeeding can protect my baby against COVID-19 and other respiratory infections.	1	0	0
COVID-19 infection in my household is an important reason to switch from breastfeeding to formula feeding.	0	0	1
**Practice part (Total scores = 6)**	**Always (point)**	**Usually or rarely (point)**	**Never (point)**
How often do you wear a mask/face shield during feeding time?	2	1	0
How often do you wash your hands with soap and water or hand sanitizer before feeding your baby?	2	1	0
How often do you wear a mask at home?	2	1	0

[1]A longitudinal survey of Thai mothers who live in Bangkok was conducted at infant age of 2,12, and 24 weeks from March 2022-April 2023 during COVID-19 pandemic

[2]The questionnaire, assessing knowledge of SARS-CoV-2 transmission, attitudes toward breastfeeding, and hygiene practices, was validated using the Item-Objective Congruence index. Good KAP was defined as scoring >60% in each section. Associated factors were analyzed using multivariable linear regression.

Supplemental Table 2 Factors associated with the change in knowledge scores from 2 to 24 weeks[1]

Factors	from 2 to 12 weeks			from 12 to 24 weeks		
	β	95% CI	*p*-value[2]	β	95%CI	*p*-value[2]
**Constant**		-0.05, 3.40	0.06		-2.62, 1.37	0.54
**Maternal age (years)**	-0.18	-0.10, -0.00	**0.04***	0.12	-0.02, 0.10	0.19
**Mother education**						
Primary school or below	Ref.	Ref.	Ref.	Ref.	Ref.	Ref.
Secondary school	-0.13	-1.67, 0.69	0.41	0.01	-1.32, 1.42	0.95
Trade/vocational school	-0.10	-1.68, 0.80	0.49	0.12	-0.84, 2.02	0.42
Bachelor's degree	0.04	-1.11, 1.37	0.84	-0.063	-1.70, 1.19	0.73
Master's degree	0.09	-1.12, 2.35	0.49	-0.18	-2.86, 1.16	0.40
Professional or doctorate	0.03	-3.09, 4.37	0.73	0.17	0.19, 8.53	**0.04***
**Mother Occupation**						
Not working	Ref.	Ref.	Ref.	Ref.	Ref.	Ref.
Working	-0.13	-1.24, 0.20	0.16	0.06	-0.55, 1.10	0.51
**Family income (USD/month)**						
< 440	Ref.	Ref.	Ref.	Ref.	Ref.	Ref.
440-880	0.03	-0.69, 0.90	0.79	-0.12	-1.39, 0.40	0.28
>880-1470	0.08	-0.54, 1.20	0.46	-0.02	-1.10, 0.90	0.85
>1470-2930	0.04	-0.83, 1.17	0.73	0.03	-0.98, 1.27	0.80
> 2930	0.06	-0.99, 1.75	0.59	-0.02	-1.76, 1.48	0.86
**Breastfeeding experience**						
No	Ref.	Ref.	Ref.	Ref.	Ref.	Ref.
Yes	0.01	-0.55, 0.61	0.91	-0.01	-0.70, 0.65	0.94
**Maternal history of COVID-19 infection**						
No	Ref.	Ref.	Ref.	Ref.	Ref.	Ref.
Yes	0.09	-0.24, 0.87	0.26	-0.10	-1.05, 0.23	0.20

Abbreviations: β: Standardized regression coefficient, CI: confidence interval, Ref: reference

[1]A longitudinal survey of Thai mothers who live in Bangkok was conducted at infant age of 2,12, and 24 weeks from March 2022-April 2023 during COVID-19 pandemic

[2]The association between change in knowledge scores and associated factors were explored using multivariable linear regression.

Supplemental Table 3 Factors associated with the change in attitude scores from 2 to 24 weeks[1]

Factors	from 2 to 12 weeks			from 12 to 24 weeks		
	**β**	**95% CI**	***p*-value[2]**	**β**	**95% CI**	***p*-value[2]**
**Constant**		-5.42, 2.09	0.38		-6.50, -0.17	0.04
**Maternal age (years)**	-0.13	-0.19, 0.03	0.14	0.186	0.02, 0.20	**0.02***
**Mother education**						
Primary school or below	Ref.	Ref.	Ref.	Ref.	Ref.	Ref.
Secondary school	0.18	-1.03, 4.12	0.24	-0.20	-3.78, 0.56	0.15
Trade/vocational school	0.30	0.21, 5.61	0.04	-0.24	-4.45, 0.11	**0.06***
Bachelor's degree	0.340	0.06, 5.47	0.05	-0.41	-5.344, -0.78	**0.01***
Master's degree	0.340	1.31, 8.89	**0.01***	-0.36	-8.11, -1.72	**<0.001***
Professional or doctorate	0.09	-3.65, 12.61	0.29	-0.20	-11.90, 1.82	0.15
**Mother Occupation**						
Not working	Ref.	Ref.	Ref.	Ref.	Ref.	Ref.
Working	-0.06	-2.15, 1.02	0.48	-0.01	-1.40, 1.27	0.93
**Family income (USD/month)**						
< 440	Ref.	Ref.	Ref.	Ref.	Ref.	Ref.
440-880	0.16	-0.43, 3.02	0.14	-0.25	-3.36, -0.45	**0.01***
>880-1470	0.25	0.45, 4.25	**0.02***	-0.41	-5.14, -1.93	**<0.001***
>1470-2930	0.15	-0.57, 3.77	0.15	-0.29	-4.62, -0.97	**<0.001***
> 2930	0.16	-0.60, 5.39	0.12	-0.35	-7.36, -2.30	**<0.001***
**Breastfeeding experience**						
No	Ref.	Ref.	Ref.	Ref.	Ref.	Ref.
Yes	0.10	-0.47, 2.06	0.22	-0.12	-1.92, 0.21	0.11
**Maternal history of COVID-19 infection**						
No	Ref.	Ref.	Ref.	Ref.	Ref.	Ref.
Yes	0.04	-0.89, 1.52	0.60	0.00	-1.02, 1.02	1.00

Abbreviations: β: Standardized regression coefficient, CI: confidence interval, Ref: reference
[1]A longitudinal survey of Thai mothers who live in Bangkok was conducted at infant age of 2,12, and 24 weeks from March 2022-April 2023 during COVID-19 pandemic
[2] The association between change in attitude scores and associated factors were explored using multivariable linear regression. *Indicates a p-value < 0.05

Supplemental Table 4 Factors associated with the change in practice scores from 2 to 24 weeks[1]

Factors	from 2 to 12 weeks			from 12 to 24 weeks		
	**β**	**95% CI**	***p*-value[2]**	**β**	**95% CI**	***p*-value[2]**
**Constant**		-2.24, 1.44	0.67		-1.16, 2.30	0.52
**Maternal age (years)**	0.11	-0.02, 0.09	0.23	-0.17	-0.09, 0.00	0.08
**Mother education**						
Primary school or below	Ref.	Ref.	Ref.	Ref.	Ref.	Ref.
Secondary school	0.08	-0.95, 1.57	0.63	0.40	0.22, 2.62	0.02
Trade/vocational school	0.01	-1.30, 1.35	0.97	0.14	-0.70, 1.79	0.39
Bachelor's degree	0.11	-0.91, 1.75	0.53	0.40	0.07, 2.55	0.04
Master's degree	0.10	-1.13, 2.58	0.44	0.05	-1.37, 1.94	0.74
Professional or doctorate	-0.03	-4.78, 3.19	0.69	-0.04	-3.44, 3.26	0.96
**Mother Occupation**						
Not working	Ref.	Ref.	Ref.	Ref.	Ref.	Ref.
Working	0.01	-0.72, 0.83	0.89	-0.06	-0.88, 0.46	0.54
**Family income (USD/month)**						
< 440	Ref.	Ref.	Ref.	Ref.	Ref.	Ref.
440-880	-0.09	-1.20, 0.49	0.41	-0.11	-1.12, 0.36	0.31
>880-1470	-0.23	-1.97, -0.10	**0.03***	-0.05	-1.00, 0.61	0.63
>1470-2930	-0.15	-1.81, 0.31	0.16	-0.05	-1.14, 0.69	0.62
> 2930	-0.18	-2.75, 0.19	0.09	0.12	-0.56, 2.03	0.26
**Breastfeeding experience**						
No	Ref.	Ref.	Ref.	Ref.	Ref.	Ref.
Yes	-0.00	-0.63, 0.60	0.96	-0.04	-0.66, 0.43	0.68
**Maternal history of COVID-19 infection**						
No	Ref.	Ref.	Ref.	Ref.	Ref.	Ref.
Yes	0.04	-0.42, 0.76	0.58	0.02	-0.45, 0.58	0.80

Abbreviations: β: Standardized regression coefficient, CI: confidence interval, Ref: reference

[1]A longitudinal survey of Thai mothers who live in Bangkok was conducted at infant age of 2,12, and 24 weeks from March 2022-April 2023 during COVID-19 pandemic

[2] The association between change in practice scores and associated factors were explored using multivariable linear regression. *Indicates a p-value < 0.05

© 2025 Ananta P. et al.
